# Exploring acceptability of oral PrEP prior to implementation among female sex workers in South Africa

**DOI:** 10.1002/jia2.25081

**Published:** 2018-02-19

**Authors:** Robyn Eakle, Adam Bourne, Judie Mbogua, Nyaradzo Mutanha, Helen Rees

**Affiliations:** ^1^ Wits Reproductive Health and HIV Institute Faculty of Health Sciences University of the Witwatersrand Johannesburg South Africa; ^2^ Social and Mathematical Epidemiology (SaME) Department of Global Health and Development London School of Hygiene and Tropical Medicine London United Kingdom; ^3^ Sigma Research Department of Social and Environmental Health Research London School of Hygiene and Tropical Medicine London United Kingdom; ^4^ Australian Research Centre in Sex, Health & Society La Trobe University Melbourne Vic. Australia; ^5^ Department of Infectious Disease Epidemiology London School of Hygiene and Tropical Medicine London United Kingdom

**Keywords:** HIV prevention, biomedical prevention products, pre‐exposure prophylaxis (PrEP), sex work, qualitative research

## Abstract

**Introduction:**

Female sex workers (FSWs) are at high‐risk for HIV acquisition in South Africa, where the advent of new HIV prevention and treatment interventions represent the potential to significantly impact the epidemic. This paper focuses on aspects of PrEP acceptability as a new intervention within the context of a larger service delivery programme including the simultaneous rollout of early ART. This paper explores PrEP acceptability among the FGD participants as future potential users.

**Methods:**

FGDs were conducted in two clinic‐based sites in Johannesburg and Pretoria. They aimed to explore community‐level, multi‐dimensional acceptability of PrEP within the context of imminent implementation alongside early ART in the TAPS Demonstration Project. Sex worker peer educators recruited participants from varying sex work locales. Facilitation was in English with adaptation by facilitators into local languages as needed. Transcripts were translated and transcribed into English. Thematic analysis was used to analyse the data.

**Results:**

Four FGDs were conducted in each site for a total of eight FDGs and 69 participants. Demographics were largely similar across the sites. Overall, there was strong acceptability of PrEP among participants and positive anticipation for the imminent delivery of PrEP in the local sex worker clinics. Themes arising from the discussions exploring aspects of PrEP acceptability included: awareness and understanding of PrEP; PrEP motivations including choice, control, and vulnerability, managing PrEP risks and worries; and, de‐stigmatizing and empowering PrEP delivery. Participant discussions and recommendations highlighted the importance of developing clear education and messaging to accurately convey the concept of PrEP, and intervention integration into supportive and tailored services.

**Conclusions:**

Through the course of these FGDs, PrEP became a positive and highly anticipated prevention option among the FSWs participants who endorsed implementation in their communities. Effective integration of PrEP into existing services will include comprehensive health programming where ART is also available, appropriate messaging, and support.

## 1 Introduction

Female sex workers (FSWs) are at high‐risk for HIV acquisition in South Africa, with a recent study reporting a prevalence of 72% in the greater Johannesburg area, and 40% and 54% in Cape Town and Durban respectively 1. The advent through clinical trials of oral pre‐exposure prophylaxis (PrEP) for HIV prevention 2, and test and treat (also “early ART”) for HIV treatment 3, 4, represent the potential to significantly impact the epidemic. As a result, these two interventions have become part of the standard of care set out by the World Health Organization (WHO) in 2015 5.

Both PrEP and early ART require consistent use (adherence) to their respective regimens to successfully prevent HIV acquisition or transmission. PrEP trials including only women struggled with maintaining adherence levels required to confer protection 6, 7, 8. As countries move towards implementation and scale‐up and in the light of ensuring proper programming is in place to support proper, consistent use, formative research on the feasibility and acceptability of integrating PrEP (and test and treat) into local and national programmes is progressing, in particular for target populations 9. However, little has yet been published in low and middle‐income country settings about community perspectives on the acceptability these interventions in a context where they are soon to be implemented. The acceptability of, and willingness to engage with, an intervention is central to its development, rollout and scale‐up, and understanding how it is may be perceived by target populations can help counter or mitigate unintended consequences or potential barriers 10, 11.

Dimensions of acceptability contributing to successful uptake and use can include personal preferences around ease of use and product attributes (including actual and perceived efficacy); social factors including stigma from partners, family, and friends, and social norms; interactions with medical facilities and service delivery mechanisms; the policy and regulatory landscape; and the context of the HIV epidemic itself 12, 13. The relative value of these dimensions is often mediated by interactions with the other dimensions as well as an individual's perception of HIV risk 13. Some of these elements were explored in previous PrEP and vaginal microbicide efficacy trials, though they more often focused on product attributes 14. Following these qualitative findings, however, a holistic, multi‐dimensional approach to acceptability was considered to be critical to promote effective use during scale‐up 15.

There is limited previously published qualitative research regarding PrEP acceptability specifically among FSWs in sub‐Saharan Africa. Most microbicide trials targeted women at high risk, which included but did not focus on sex workers 16, 17, 18, 19, 20, apart from some of the nonoxynol‐9 vaginal gel studies 21, 22. One study on intermittent PrEP in Kenya included men who have sex with men (MSM) and FSWs 23, however, only five FSWs were included and results were not disaggregated. Lenses into PrEP acceptability among sex workers have been made available through research with people who use drugs, such as one Canadian study which found that engaging in sex work was in itself a motivation to take up PrEP 24. While limited in scope, findings from these studies, have pointed to values and perspectives around PrEP particular to FSWs' work and roles as women.

Given that the transition to PrEP implementation is recent, the multiple dimensions of acceptability are yet to be fully understood as drug‐based prevention is unfamiliar to the wider public. In addition, service providers' experience with rolling out ART programmes, and the delivery of contraception to prevent unwanted pregnancies, could provide a basis for understanding PrEP where services would be similar, but motivations to take the medication would differ in prevention versus treatment.

In this paper, we present findings from focus group discussions (FGDs) that sought to inform the design of oral PrEP and (then called) early ART interventions for FSWs in Johannesburg and Pretoria for implementation in the Treatment And Prevention for Sex workers (TAPS) Demonstration Project 25. The findings presented here focus solely on dimensions of PrEP acceptability, where at the time of data collection, the role and potential of PrEP had not yet been normalized by government guidelines and national regulatory approval. This paper considers PrEP as a new intervention within the context of impending provisions within a larger service delivery programme including the simultaneous rollout of early ART. The aim of this paper is to specifically explore PrEP acceptability among the FGD participants as future potential users.

## 2 Methods

FGDs were conducted in two clinic‐based settings in Johannesburg and Pretoria between July and December 2014. The two clinics were part of the larger Wits RHI Sex Worker Programme which provides comprehensive primary care services to female, male and transgender sex workers in a variety of settings. These services include: HIV counselling and testing and condom distribution, NIMART (nurse‐initiated and managed antiretroviral treatment), tuberculosis screening, HPV screening, clinical services for minor ailments, psychosocial support, and referrals to both clinical and legal services. The clinics, services and programme are described in further detail in several other publications, including a detailed account of the formative research conducted during the design of the TAPS project 25, 26, 27, 28, 29.

They aimed to explore community‐level acceptability of a PrEP (and early ART) intervention with a view to learn how this could influence uptake and use in TAPS. A holistic, multi‐dimensional approach to acceptability was employed, including perspectives from both HIV‐negative and HIV‐positive women on both interventions.

Initial topics for discussion were developed based on the results of an adapted meta‐ethnography of women's experiences of uptake and use of biomedical HIV prevention products across sub‐Saharan Africa 30, 31, as well as the Modified Social Ecological Model (MSEM) designed by Baral et al. 32. Discussions encouraged community‐level narratives of women's lives and experiences with their work and health services; perceptions of optimal prevention and treatment delivery; knowledge, attitudes, and perceptions of health and wellbeing, and of PrEP and early ART specifically; acceptability of and commitment to increased, frequent clinical monitoring and HIV testing; and identifying overall motivations and barriers to accessing services, and in particular, PrEP as the newest intervention.

Sex worker peer educators recruited women through their work/social networks from varying sex work locales (brothels, streets and other informal areas) aiming to gather diverse perspectives from different working and living environments. Only cis gendered women were recruited as the eventual demonstration project did not include any other populations. Discussions were facilitated primarily in English with adaptation by facilitators and staff note takers into local languages as needed. An introduction was provided at the beginning of each FGD session to orient participants as to the purpose of the discussions and the topics to be discussed. This is included in Annex 1. Background information on PrEP (and early ART) was provided within the context of each group dynamic and as the topic arose, which varied depending on participant's knowledge. FGDs lasted between one and a half and two hours. Participants in the FGDs were given R50 (~4 USD) to reimburse for travel costs and refreshments in the form of juice and sandwiches following each FGD session. No other compensation was given for this research.

### 2.1 Analysis

Transcripts for each of the FGDs were translated and transcribed into English, then uploaded into NVIVO (version 10.0, Burlington MA). Analysis followed principles of Braun and Clarke's thematic analysis 33 beginning with a set of questions building from themes derived from the discussion guide based on the MSEM but we remained open to new lines of enquiry as they emerged from the data in an inductive manner. The coding framework was devised by the primary author with support from two other researchers, and all transcripts were coded in duplicate by two researchers. Inter‐coder reliability was not a focus of the analysis, rather coding choices were discussed throughout and at the end of the coding process to resolve any differences.

### 2.2 Ethical considerations

All participants provided their informed consent and pseudonyms to protect identities, indicated in this paper as colours. This study was reviewed and approved by the Wits Human Research Ethics Committee (reference number: M131009).

## 3 Results

### 3.1 Participant demographics

Four FGDs were conducted in each site for a total of eight FDGs and 69 participants. Key demographic characteristics are displayed in Table 1.

**Table 1:** Demographic characteristics of FGD participants

Characteristics	Hillbrow n = 39	Pretoria n = 30	Total
Age			
20 to 29	8	15	23
30 to 44	26	12	38
45+	5	3	8
Relationship status			
Single	35	24	59
Married or steady partner	1	2	3
Divorced or separated	2	2	4
Widowed	1	2	3
Education			
No School	0	2	2
Primary	3	1	4
Grade 8 to 12	35	25	60
Higher	1	2	3
Place of Birth^a^			
Gauteng	7	13	20
Other SA Provinces	22	6	28
Other Countries	10	10	20
Place of Residence (by area)			
Hillbrow	20		20
Greater Johannesburg	19	3	22
Pretoria CBD		15	15
Greater Pretoria		10	10
Brits (Northwest Province)		2	2

Other SA provinces, Free State, Eastern Cape, KwaZulu‐Natal, Limpopo, Mpumalanga; Other Countries, Zimbabwe, Mozambique, Lesotho; Greater Johannesburg, Central Business District (CBD), Soweto, Yeoville, Berea, Ekurhuleni; Greater Pretoria, Soshanguve, Mamelodi, Atteridgeville.

a 1 participant did not list place of birth in Pretoria.

Participants were not asked to disclose their HIV status, however most participants did so of their own volition during the discussions. From their disclosures we estimate about 50% to 75% of the participants were HIV‐positive, which is similar to the estimated prevalence in the greater Johannesburg area 1, and to what was observed in Pretoria during outreach and HIV testing during formative work.

While there was relative diversity among the FGD participants, themes arising from the discussions did not differ across the groups. To qualitatively assess overall aspects of PrEP acceptability, we review the thematic findings here in terms of: awareness and understanding of PrEP; PrEP motivations including choice, control, and vulnerability, managing PrEP risks and worries; and, de‐stigmatizing and empowering PrEP delivery. Quotes from participants are labelled with their chosen colour pseudonyms, abbreviation of site location (JHB=Johannesburg; PTA= Pretoria), and FGD number.

### 3.2 Awareness and understanding of PrEP

Prior awareness of PrEP was explored within the context of existing or known HIV prevention options. Most of the discussion centred on male and female condoms, however some women acknowledged male circumcision and prevention of mother to child transmission (PMTCT), and some had heard of, or participated in, studies involving the vaginal gel and ring. Apart from condoms, post‐exposure prophylaxis (PEP) was most often discussed, along with STI treatments and/or the morning after pill for unwanted pregnancies. “If you do sleep with a client and a condom gets broken …. everybody decides to drink that pill. But it's just for cleaning, we know it doesn't prevent HIV” (Brown, PTA 3). Considering PrEP within the context of other previously known options, or lack thereof, allowed women to recognize and interpret PrEP as a potential choice for prevention.

Prior to a detailed description of PrEP within the FGDs, awareness was low. Initial understanding of PrEP was usually reached by equating it with contraception. “Okay meaning that people who are going to take this tablet they must be very careful because a pill for preventing, it's very risky, because once you forget …. like on birth control pills, it's very risky ….” (Red, PTA 4). Alternatively, the concept of PrEP was further assimilated through prior knowledge and experience with HIV testing and ART.

Questions relating to awareness and understanding of PrEP frequently resulted in discussions about what type of woman could and should use PrEP, or who would make a “good PrEP taker.” There was universal understanding of motivations to take PrEP for HIV‐negative women, however it was the need for a certain degree of commitment which the participants focused on:

She has to take care for herself …., so she must behave like a person who prevents when she is avoiding to fall pregnant. But she shouldn't be like when someone prevents and they skip a pill and they would say “aggh even if I forgot to take my pill nothing will happen to me.” So with PrEP she must continue using it every day (Green, PTA 4).

### 3.3 PrEP motivations: choice, control and vulnerability

An awareness of the high prevalence of HIV among FSWs in their communities underscored the need for more prevention options to safeguard those who were still HIV‐negative. “The methods are very limited. …. The only method that we know so far is to use a condom, and…. it can burst anytime” (Brown, JHB 1).

These sentiments laid the groundwork for seemingly universal approval and anticipation of PrEP, which FGD facilitators indicated would be imminently offered in the clinics in their locality:

People are dying outside of HIV. And HIV is spreading so fast …. At least people who are still negative will have a chance to stay negative…. So the spreading of HIV will get limited. Then as time goes by the HIV rate will go down …. I don't want my daughter to go through what I am going through now. (Brown, JHB 1).

All of the women immediately saw the benefit of having PrEP in the body, especially given that condoms often burst or were removed. *“*PrEP is good because it stays in your blood*”* (Red, PTA 3). As sex workers, participants reported how they frequently found themselves in vulnerable positions with clients, which could be hard to anticipate or plan for. In such circumstances, PrEP could be invaluable in protecting women from HIV where they would have personal control over one method of protection. “Even with a client in your own house, they can throw away the condom, strangle you and leave you helpless because you do not want to use a condom. And do as they please with you*”* (Purple, JHB1).

Experiences of sexual violence were frequently reported (unprompted) within groups, and were seen as particularly motivating for PrEP uptake and use. “Another thing, as a sex worker, there is no one here who say they not been gilwa (treated badly). Maybe they even, some of us we have even been ganged raped. So I believe we have to test continuously, time and again” (Brown, JHB 1).

Finally, in their own recognition that the primary HIV risk for FSWs came from their main partners rather than clients, many participants reflected on the utility of PrEP in protecting them in the likely event that their partner was also having sex with other women:

Even if she says, that boyfriend, she loves him, there are many hotels. He doesn't have her only. The other hotels there are many he's got another girlfriend, the other hotel, he's got another. And he's been sleeping with them without that condom (Red, JHB1).

While at the time of the FGDs many of the women did not report having a current main partner, most of the women recounted experiences with previous main partners which they felt primarily contributed to their risk (and often led to the end of those partnerships). Such sentiment informed a belief expressed by many that PrEP should be made available to all women, rather than just FSWs. “Yes I also agree with them that the entire woman should use PrEP who are HIV‐negative because again they also don't know who their husbands are sleeping with” (Brown, PTA, 4).

### 3.4 Managing PrEP risks and worries

While the potential to reduce HIV transmission was keenly recognized and appreciated, women were also acutely aware of a range of other risks in their sexual, social and personal environments that might influence their willingness to use PrEP. These would, they felt, require careful consideration and management.

A primary concern related to the potential discontinuation of condom use in the context of PrEP, and the implications of this for acquisition of other sexually transmitted infections (STIs) or unwanted pregnancies:

But what I think, neh, if they are going to use these PrEP pills they have to teach people well, that they must use condoms. Make them to be aware of what is going to happen because of, if they say ok you can keep on drinking these pills they will start forgetting to use condoms and start to sleep around without. That's why they're scared, that many teenagers of South Africa, they will have plenty of babies (Red, JHB 1).

All of the women agreed that PrEP could not take the place of condoms, and that the two should go together in order to prevent other STIs and unwanted pregnancies, especially since most of the women acknowledged not using other modes of contraception. This perception reflected a general sense that PrEP would serve as useful backup when condoms failed, rather than being seen as the primary prevention strategy.

The risk of potential non‐adherence, and thus diminished PrEP effectiveness, was commonly expressed. Many participants felt that some women may not be sufficiently motivated to take a pill every day, especially when they were not feeling unwell. This experience was reflected by some in the group who disclosed their HIV‐positive status:

It's difficult to support somebody who's not sick and say she must take medication especially every day. At the clinic they will always check and tell you that your viral load is high, but you will tell yourself that there is no need to take that medication, rather take drugs and you will be ok (Brown, PTA 3).

Additional worries around adherence included substance use (alcohol and illicit drugs) and the potential for forgetting to take a daily pill. “…. those who are using drugs, they won't be able to take PrEP every day because they will forget under the influence of drugs” (Brown, PTA, 3). However, there was also discussion about how lessons learned in ART adherence among HIV‐positive women could help improve adherence among those taking PrEP, such as aligning pill taking with television shows, using phone alarms, and coming up with strategies to keep pills on hand such as in secret bra compartments.

Importantly, the discussion of substance use raised questions about whether PrEP could be taken even when someone knew they would be drinking or using other substances during the course of the day. “So this PrEP it's not for HIV‐positive, so I want to ask something, is there somewhere where it says don't smoke, don't drink ….?” (Grey, PTA 4). Explaining that using alcohol or other drugs would not diminish the preventive effects of PrEP was met with approval from the participants.

The perception of potential side effects, especially given knowledge of ART side effects from existing drugs on the market, was seen as a likely major barrier to uptake:

Yes, maybe they will come for counselling first before they take the PrEP right, and then they tell them about the side effects and then a person gets scared of it and say, my goodness no, you know what these side effects are going to do to me, “they will make me sick, they will make me throw up, I will die” you see that kind of thing, or you will be paining (Red, JHB 4).

Interestingly, as a result, participants felt it would be important to ensure clear messaging around potential side effects and their duration was central to in the promotion of PrEP.

Perhaps less common were concerns that focused more on logistical and social aspects outside of the women's control, such as the pills being stolen outside clinics:

They know that this one has come to fetch her pills, they steal them from you, you get hurt. They know that they are going to use the pills as drugs. You know that you will be mugged, they hold you up they stab you, what do you do? You are now not killed because of your AIDS but for your pills (Purple, JHB 1).

### 3.5 De‐stigmatizing and empowering PrEP delivery

The need for supportive, non‐judgmental services tailored to sex workers was universally stated. “It is right that such a clinic is there. …. having such a clinic will make people not shy to go to the clinic. They can speak freely, because the clinic is ours” (Black, JHB 4). Indeed, positive interactions in the clinic would motivate women to return: “….when a clinic treats you well, you return to it, but if they don't treat you properly you don't return” (Red, JHB 2). Including PrEP as part of this type of service delivery environment was considered paramount to successful implementation.

In addition, having PrEP offered alongside early ART was considered important in destigmatizing interventions. Given the high HIV prevalence rate, of which women were keenly aware, there was a sense of chronic distress concerning the inevitability of becoming HIV‐positive among the larger FSW population, and certainly among the FGD participants. Therefore, many of the women felt that PrEP presented an opportunity to “de‐stress”, or remove some of that panic and the dread of HIV infection. In addition, the HIV‐positive women felt that having additional protection in the community might result in fewer infections.

The need for social support, often initially voiced by women with ART experiences, was identified universally as a valued component of pill‐taking and a way to help ensure commitment:

If you get somebody who supports you in what you are doing it's much more easier for you to continue than when they don't have anybody next to you. Like a person like my sister is my biggest supporter. Immediately when I found out I am positive that's the first person I told. (Purple, JHB 1).

Finally, flexibility in service delivery was underscored as critical for a successful PrEP intervention. This stemmed from not having much time to go to the clinic due to having to work long hours to pay rent, as well as having become habituated to mobile services in the case of the Johannesburg groups:

I don't pay rent. I do business to feed myself. The other's on the streets they are staying in flats.… they need [money] for rent that's why they work the whole night. So we are different in that, you who is staying in a hotel, you don't have time to come to clinic, you are waiting for a mobile clinic to come, you understand? (Green, JHB 1).

## 4 Discussion

Our findings indicate strong acceptability of PrEP among FSWs in the two communities and positive anticipation for the imminent delivery of PrEP in the local sex worker clinics. Indeed, women were so positive at the end of each session that they independently asked to create waiting lists to be informed as soon as the TAPS project was launched so they could be the first to enrol. These FGD participants in turn provided useful insights according to their concerns into how uptake and use could be ensured by this most‐at‐risk population in South Africa. These centred on developing clear education and messaging to accurately convey the concept of PrEP and the need to ensure continued condom use, as well as intervention integration into supportive and tailored services.

Fitting PrEP within existing knowledge of ART and PEP, while equating it with contraception as a prevention modality, was an effective and naturally developed education strategy among participants, which has also been true among researchers 34. In addition, experiences with early ART or PEP can translate to PrEP, such as taking medication every day even while feeling well. Discussions in these FGDs suggested that HIV‐negative and positive women could provide valuable support systems, a strategy which was successfully used in another study in Zimbabwe 35, and may prove useful for normalizing and implementing the two interventions in scale‐up. Since the TAPS project was to be implemented in the largely peer‐driven health services of the Wits RHI Sex Worker Programme, the interest from HIV‐positive women as well as older sex workers generally, presented an opportunity to consider additional support and normalization systems.

Discussions concerning condom use usually focused on the reliability of condoms with clients (where clients could not be trusted not to remove or damage condoms, or condoms themselves could not be trusted not to burst), and use with main partners with whom condom use was seen as untrusting or unloving. This translated into one of the key motivations for use of having an additional layer of protection and personal control, to safeguard against the unpredictable such as sexual violence at work or lack of condom use with main partners with an unknown HIV status. This notion of additional protection and the relevant reasons are fairly different than what has been articulated by MSM who have been more concerned with (lack of) accurate risk perception and stigma within their social circles 36, suggesting the need to carefully consider differences in population perspectives when planning to rollout PrEP. Interestingly, changes in condom use over time were primarily seen in the efficacy and implementation studies with MSM, as opposed to the studies with heterosexual men and women 2 which may correlate with the different needs and desires of the populations.

It should also be noted that many of the women in these FGDs declared not currently using contraception other than condoms. This is reflected in other studies conducted in the same area 1, 28. This is largely due to the lack of availability of options outside of the Depo‐provera injection which was known in the population to have side effects (cramps, excessive bleeding, nausea) which negated the contraceptive benefit, especially in the context of the work environment. While this was not explored in detail in this particular research, the reasons behind lack of contraception use were well‐known in the community and the larger health programme which was in the process of implementing additional contraception options at the time these FGDs were conducted. In addition, considering the implementation aspects of PrEP could also be an opportunity to expand contraception options and improve service delivery, as well for other relevant health needs such as STIs.

Of particular note were the participants' worries and concerns in our study about the broader effects of PrEP and potential for misuse and misinterpretation. Their recognition, combined with the same awareness among other current and existing users in other parts of the world 36, 37, 38, should come as a reassurance to those with apprehensions that the introduction of PrEP could be mismanaged leading to declines in condom use 39. Participants felt strongly that the addition of PrEP should be seen as a back‐up for when condoms failed for the various reasons previously mentioned, but that PrEP was an important new option that should be offered to all HIV‐negative women.

Finally, data from our study suggest that normalization may be an important factor when considering PrEP rollout. There was overall agreement that all women, and even men, should have access to PrEP, and that PrEP should be combined with early ART in the same clinic. The notion of stress around HIV infection was a strong recurring theme, relating to individual disease burden as well as stigma, where a strategy around normalization of both PrEP and early ART could play an important role. The articulation of the stress of HIV and stigmatization of pill taking, points as well to the need to include HIV‐positive individuals in the discussion, to garner their perspectives and longer‐term commitment to pill taking.

The main limitation in this research is the snowball sampling used to recruit participants which may have resulted in enrolment of more informed or vocal women and/or women who have been continuously involved in sex worker programme outreach activities. However, this was less the case in Pretoria where services had not previously existed, and these women may have represented potential “early adopters” who could be critical in promoting PrEP. There were also fewer women who had a main partner at the time of the sessions than seen in the larger local population 1, 28. In addition, the FGDs included only FSWs and took place only in two urban areas, which may limit generalizability to other populations and contexts. The theoretical nature of the FGDs, where actual use was imminent but not yet in place, may not translate into subsequent uptake of PrEP. Finally, the overwhelming positivity towards PrEP (and early ART) as an outcome of the FGDs was surprising. Negative sentiments were actively probed during the sessions but were difficult to draw out. It is possible that this reflects the group setting and a conformity bias, though participants' suggestions to start waiting lists for the intervention launch was unprompted, indicating high and independent positivity.

## 5 Conclusion

Through the course of these FGDs, PrEP became a highly anticipated prevention option among the FSWs participants who positively endorsed its implementation in their communities. Participants shared important insights and interpretations of acceptability for consideration in implementation, including the imperative for nuanced messaging around condom use and risk reduction. Integrating PrEP into existing services as part of a comprehensive health programme, where ART is also available, was seen as a best practice as long as attention is paid to ensuring the appropriate messaging and support are included.

## Competing interests

The authors have no declarations or conflicts of interest associated with this work.

## Authors' contributions

RE designed the study and data collection tools, participated in data collection, analysed the data and drafted the paper. AB supported study and data collection tool design and data analysis. JM facilitated data collection and analysis. NM supported data collection. HR oversaw the project as Principal Investigator. All authors contributed to drafting and finalizing the manuscript.

## Funding

This research was funded by AIDS Fonds Netherlands and the Bill and Melinda Gates Foundation (grant OPP1084416).

## Annex 1

### Introduction to Focus Group Discussions for Participants

The following text was read at the start of each focus group discussion session to orient participants as to the purpose of the discussions and topics to be discussed. We are holding a few discussions with up to 10 women at a time to talk about experiences with health care in general and more specifically HIV testing, prevention and care services. These discussions will help us understand whether we can put together a set of services tailored especially for female sex workers that will support their needs. We would like to find out what you think about current services, what is missing from them, what kinds of services you wish were available, difficulties in accessing testing and care, and your thoughts about new potential services.
